# Functions of *N*-Glycosylation-Related Endoplasmic Reticulum Proteins in the Development and Virulence of Plant Pathogens

**DOI:** 10.3390/jof11110791

**Published:** 2025-11-05

**Authors:** Yanxin Wang, Kaijie Chen, Yu Zhang, Zimeng Zhang, Zi Tao, Xianfeng Ye

**Affiliations:** 1 College of Agriculture and Biology, Liaocheng University, Liaocheng 252000, China; 2 Key Laboratory of Agricultural Environmental Microbiology, Ministry of Agriculture and Rural Affairs, College of Life Sciences, Nanjing Agricultural University, Nanjing 210095, China

**Keywords:** *N*-glycosylation, endoplasmic reticulum, plant pathogens, development, pathogenicity

## Abstract

*N*-glycosylation, a crucial eukaryotic post-translational modification, has been extensively studied for its significance in the physiology and virulence processes of plant pathogens over the last decade. This review systematically analyzes the functions of *N*-glycosylation-related endoplasmic reticulum (ER) proteins in regulating plant pathogen processes, including mycelial growth, conidial development, host penetration as well as colonization, pathogenicity, cell wall integrity and host immune evasion. By modifying the structure and function of target proteins, these ER-localized proteins regulate essential developmental events in pathogens while concurrently mediating interactions between pathogens and plants, influencing pathogens’ growth and disease-causing potential. Future research requires the systematic delineation of glycosylation-regulated protein networks by multi-omics integration technologies and the elucidation of their functional processes using molecular genetics methodologies, thereby establishing a robust theoretical foundation for the development of novel biological fungicides.

## 1. Introduction

Plant pathogens are a significant category of microbes that inflict damage by colonizing and infecting various plant tissues, such as roots, stems, leaves, flowers, and fruits [1]. Agricultural production is profoundly influenced by crop diseases, including rice blast caused by *Magnaporthe oryzae* [2], corn smut caused by *Ustilago maydis* [3], and fusarium wilt caused by *Fusarium oxysporum* [4,5], which markedly diminishes productivity and compromise product quality. These impacts collectively pose a substantial threat to global food security, the welfare of farmers, and the sustainable advancement of agricultural practices [6,7]. Considering the increasing global population and the consequent pressure on food resources, it is imperative to investigate the pathogenesis of these plant pathogens and to improve efficient control strategies, thereby safeguarding agricultural output and ensuring a dependable food supply for future generations.

*N*-glycosylation is a conserved post-translational modification in eukaryotic cells [8], involving the attachment of an oligosaccharide chain to the asparagine (Asn) residue of proteins containing the consensus sequence Asn-X-Ser/Thr (where X is not proline) during translation. All N-linked glycans possess a conserved pentasaccharide core structure [9]. This modification regulates protein folding, structural stability, quality control, intracellular trafficking efficiency and subcellular localization accuracy, hence influencing essential biological processes in multicellular organisms [10]. *N*-glycosylation in mammals modulates immune responses, facilitates signal transduction pathways, and supports neurodevelopmental processes [11]. In plants, it similarly regulates seed development and facilitates adaptive responses to abiotic stresses, including drought and salinity [12]. Yeast relies on *N*-glycosylation for protein secretion and the maintenance of cell wall integrity [13].

Recent scientific interest has intensified on the significance of *N*-glycosylation in phytopathogenic fungi and oomycetes. Nevertheless, investigations into its functional significance have thus far been confined to a rather small spectrum of plant pathogenic species. The growth and development of *M. oryzae*, *Colletotrichum graminicola*, and *Phytophthora sojae* are significantly hindered by tunicamycin, which specifically obstructs the Alg7-mediated first phase of N-linked glycan synthesis [14]. This suppression is evidenced by the disruption of appressorium formation and invasive hyphal elongation in *M. oryzae* and *C. graminicola* [15,16], alongside a decrease in cyst germination efficiency and oospore production in *P. sojae* [17]. Moreover, numerous proteins undergo *N*-glycosylation modifications during pathogen proliferation. Quantitative proteomic analyses have identified 559 and 496 *N*-glycosylation sites among 355 proteins in *M. oryzae* and *P. sojae*, respectively [15,17], and 676 sites among 351 proteins in *C. graminicola* [16]. This study consolidates the impact of *N*-glycosylation-related ER proteins on pathogen development and virulence, underscoring the critical significance of this post-translational modification and providing a crucial theoretical basis for the identification of novel antifungal targets.

## 2. Processes of N-Linked Glycan Assembly and Modification in ER

The *N*-glycosylation in *Saccharomyces cerevisiae* has been extensively characterized, consisting of two sequential steps, assembly and processing [18]. Given the significant homology between *N*-glycosylation modifications in plant pathogens and those in *S. cerevisiae*, researchers have employed the established *N*-glycosylation pathway and its associated key genes in *S. cerevisiae* as a reference framework. Utilizing homology alignment, these genes are mapped to the genomes of plant pathogens, followed by functional validation of their homologous counterparts within the pathogens. Based on these, the glycosyltransferases, flippase, oligosaccharyltransferases (OST) implicated in *N*-glycosylation assembly [19,20,21], as well as the glucosidases, molecular chaperones and glucosyltransferase responsible for *N*-glycosylation modification [22], along with their functions and products, are presented in Table 1. This study emphasizes the conserved post-translational modifications inside the ER, in contrast to the intricate and species-specific modifications occurring in the Golgi apparatus [23].

### 2.1. Assembly of N-Linked Glycan

During assembly, dolichyl phosphate (Dol-P), produced by the enzymatic action of dolichol kinase Sec59p on dolichol and CTP [24], serves as an essential carrier in the synthesis of lipid-linked oligosaccharides (LLO). On the cytoplasmic side of the ER, Dol-P-bound sugars, essential precursors for LLO assembly, are synthesized by the interaction of Dol-P with diverse nucleotide-activated sugars, mediated by specific synthases. Dol-P-Glc is generated via the catalytic activity of Dol-P-Glc synthase Alg5 (Alg, asparagine-linked glycosylation) on UDP-Glc [25], whereas Dol-P-Man is synthesized by Dol-P-Man synthase Dpm1p acting on GDP-Man [26]. However, the translocation mechanism of Dol-P bound sugars from the cytoplasmic side to the ER luminal side remains unexplained.

**Figure 1:**
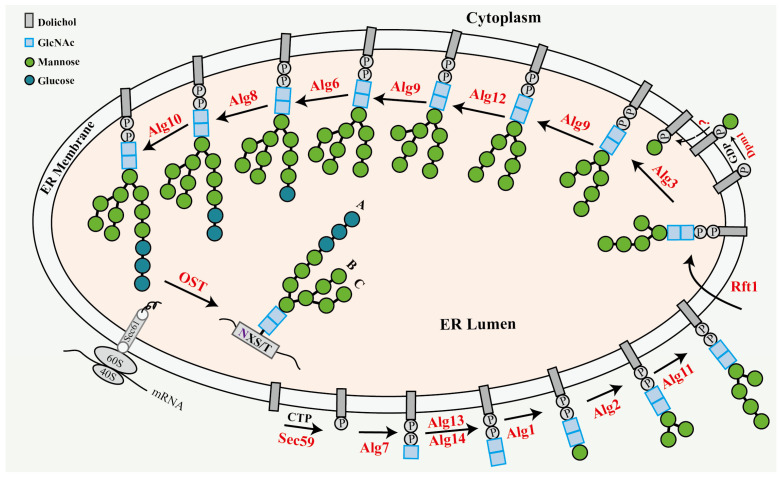
Diagram illustrating the stepwise construction of oligosaccharide chains within the endoplasmic reticulum during the *N*-glycosylation in *Saccharomyces cerevisiae*. The text in red font indicates the proteins implicated in this process.

The biosynthesis of LLO commences with the action of *N*-acetylglucosamine-phosphate transferase Alg7, which catalyzes the transfer of *N*-acetylglucosamine phosphate (GlcNAc-1-P) to Dol-P, yielding dolichylpyrophosphate-GlcNAc (Dol-PP-GlcNAc) [27]. The protein complexes Alg13/Alg14 facilitate the addition of the second GlcNAc residue, resulting in the synthesis of Dol-PP-GlcNAc_2_ [28]. Three mannosyltransferases, Alg1, Alg2, and Alg11, progressively add five mannose (Man) residues to generate the intermediate (Dol-PP-GlcNAc_2_-Man_5_). The β-1,4-mannosyltransferase Alg1 catalyzes the first attachment of β-1,4-linked mannose [29]. Thereafter, the α-1,3/α-1,6-mannosyltransferase Alg2 catalyzes the addition of two branched mannoses through α-1,3 and α-1,6-glycosidic bonds [30]. Ultimately, the α-1,2-mannosyltransferase Alg11 elongates the lipid-linked oligosaccharide (LLO) by successively incorporating two α-1,2-linked mannoses to the α-1,3-linked mannose (A-branch) [31]. Subsequently, the proposed flippase Rft1 facilitates the translocation of the intermediate from the cytoplasmic side to the ER lumen [32]. In the ER lumen, the α-1,3-mannosyltransferase Alg3 and α-1,2-mannosyltransferase Alg9 sequentially synthesize the B-branch of LLO through systematically adding α-1,3-mannose and α-1,2-mannose [33,34]. Thereafter, α-1,6-mannosyltransferase Alg12 and α-1,2-mannosyltransferase Alg9 facilitate the sequential incorporation of α-1,6 and α-1,2-linked mannose into the C-branch of LLO [35]. Ultimately, the glucosyltransferases Alg6, Alg8, and Alg10 are responsible for elongating the A-branch of LLO. Alg6 and Alg8 contribute the initial two α-1,3-linked glucoses, whereas Alg10 facilitates the addition of the third α-1,2-linked glucose [36], culminating in the formation of Dol-PP-GlcNAc_2_-Man_9_-Glc_3_, the fully assembled LLO. The oligosaccharyltransferases (OST), a multimeric complex consisting of eight protein subunits (subcomplex 1: Wbp1, Swp1, and Ost2; subcomplex 2: Stt3p, Ost3/6p, and Ost4p; subcomplex 3: Ost1 and Ost5), facilitate the transfer of preassembled oligosaccharide (Glc_3_Man_9_GlcNAc_2_) from Dol-PP carrier to the side-chain amide nitrogen of asparagine residues within the consensus sequence (asparagine-X-serine/threonine, X cannot be proline) in nascent polypeptides [9]. All previously described processes in LLO assembly are clearly illustrated in Figure 1.

### 2.2. Processing of N-Linked Glycan in ER

**Figure 2:**
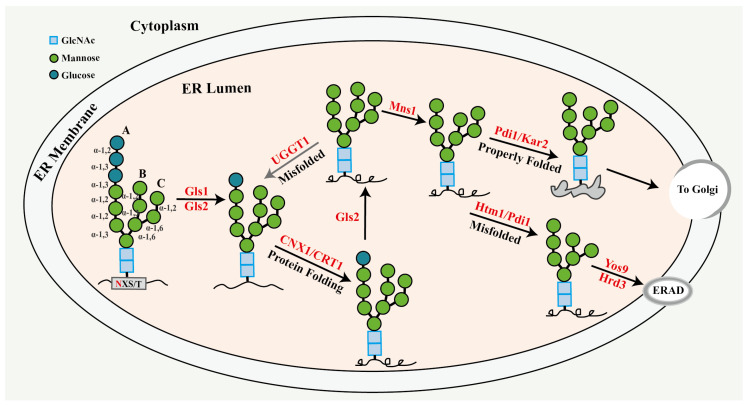
Illustrative diagram depicting the successive processing of oligosaccharide chains within the endoplasmic reticulum lumen during the *N*-glycosylation process in *Saccharomyces cerevisiae*. The text in red font indicates the proteins implicated in this process.

N-linked glycan processing initiates in the ER lumen and is closely associated with polypeptide folding [37]. Glucosidase I (GlsI) and glucosidase II (Gls2), encoded by *CWH41* and *ROT2*, respectively, initially eliminate the terminal α-1,2 and α-1,3-linked glucose residues from the A branch of the oligosaccharide precursor [13,38]. Subsequent, calnexin (CNX1) or calreticulin (CRT1) binds the monoglucosylated oligosaccharide to promote the correct folding of polypeptide chains [39], after which they dissociate upon the terminal glucose from the A branch of the oligosaccharide is captured and removed by glucosidase II. Should polypeptides persist in a non-native conformation, the UDP-Glc: glycoprotein glycosyltransferase (UGGT) re-glycosylates the A-branch of the oligosaccharide by reintroducing the glucose residue, resulting in the formation of a monoglucosylated intermediate. This intermediate subsequently binds to CNX1/CRT1, re-entering the folding cycle until the polypeptides attain the correct conformation [40]. Should the polypeptides do not achieve proper folding within the designated time, α-1,2-mannosidase I (Mns1) will excise the terminal mannose from the B branch of the oligosaccharide [41]. Should the polypeptides attain proper conformation with the aid of chaperones like Pdi1p and Kar2p [42,43], the native glycoprotein possessing the N-linked oligosaccharide chain (GlcNAc_2_Man_8_) would exit the ER. Otherwise, the protein Htm1 would recognize the N-linked oligosaccharide chain characterized by the B-branch containing a single mannose residue, subsequently cleaving the terminal mannose from the C-branch, thereby exposing the α-1,6-mannose at the terminus of the C-branch. Subsequently, the glycoprotein is recognized by the lectin (Yos9p) linked with the ER-associated degradation (ERAD) pathway, then ubiquitinated by Hrd1p, and ultimately degraded by proteasomes [44]. All of the aforementioned steps in N-linked glycan modification are illustrated in Figure 2.

## 3. *N*-Glycosylation and Plant Pathogens

*N*-Glycosylation in plant pathogens has attracted extensive attention over the past decade [45,46]; however, current comprehension of its effects on the growth, development, and pathogenicity of these microorganisms is predominantly derived from a restricted number of pathogenic species, due to the limited methodologies available for monitoring this post-translational modification during pathogen development. This work presents a comprehensive analysis of the influence of ER-associated proteins involved in *N*-glycosylation on plant pathogens, as shown in Table 2. This paper analyzes twenty-one *N*-glycosylation-associated proteins from nine plant pathogens, categorized into ten assembly-related and eleven modification-related proteins. The first category encompasses *N*-acetylglucosaminyl transferase (FoGnt2 from *F. oxysporum*), α-1,2-mannosyltransferase (MgAlg2 from *Mycosphaerella graminicola*), α-1,3-mannosyltransferases (MoAlg3 from *M. oryzae*, CgAlg3 from *C. graminicola* and UvAlg3 from *Ustilaginoidea virens*), α-1,2-mannosyltransferases (MoAlg9 from *M. oryzae*), α-1,6-mannosyltransferases (FoOch1 from *F. oxysporum f*. sp. *cubense* and VdOch1 from *Verticillium dahlia*) as well as oligosaccharyltransferases (VdSTT3 from *V. dahlia* and PcSTT3 from *P. capsici*). The second category includes glucoside I (UmGls1 from *U. maydis* and MoGls1 from *M. oryzae*), glucoside II (UmGas1 from *U. maydis* and MoGls2 from *M. oryzae*) along with its β-subunit (UmGas2 from *U. maydis* and MoGtb1 from *M. oryzae*), calnexin (UmCNX1 from *U. maydis*, MoCNX1 from *M. oryzae* and CgCNX from *C. graminicola*), disulfide isomerase (UmPdi1 from *U. maydis*) and mannosidase (UmMns1 from *U. maydi*).

### 3.1. N-Glycosylation Impacts the Hyphal Growth of Plant Pathogens

Normal vegetative growth is fundamental for effectively infecting the host during the complex lifecycle of phytopathogenic fungi. Hyphae of plant pathogens efficiently assimilate nutrients from the environment [7], facilitating further infection and colonization, as demonstrated by *Fusarium* species, which accumulates glycogen and lipids to support their invasive growth within host [59]. Moreover, they recognize surface-specific signals from host plant to precisely identify compatible hosts, as evidenced in *M. oryzae*, which detects unique sugar signals on rice leaf surfaces to initiate attachment and infection [60,61]. Furthermore, they release oxidoreductases to decompose reactive oxygen species generated by the host [62], thereby undermining the host’s chemical defenses against infections.

Considering the pivotal function of mycelia in the pathogenicity of phytopathogens, the impact of *N*-glycosylation-related proteins on mycelial proliferation has been systematically investigated. Mutational investigations have indicated that numerous critical components involved in N-linked glycan assembly are essential for hyphal growth and morphogenesis, as evidenced by phenotypic abnormalities in corresponding mutants. Specifically, the deletions of α-1,3-mannosyltransferases (MoAlg3 in *M. oryzae*, CgAlg3 in *C. graminicola*, and UvAlg3 in *U. virens*) lead to growth inhibition of 6% in *M. oryzae* [49], 29% in *C. graminicola* [16], and 30% in *U. virens* [50]. Conversely, the disruptions of α-1,6-mannosyltransferases (FoOch1 in *F. oxysporum f*. sp. *cubense* and VdOch1 in *V. dahliae*) result in substantial growth deficient, with a reduction of 60% in both species [51,52]. Mutants deficient in oligosaccharyltransferases (OST) (VdSTT3 in *V. dahliae* and PcSTT3 in *P. capsici*) exhibit a 30% reduction in growth [53,54]. Moreover, the deletion of the *N*-acetylglucosaminyl transferase FoGnt2 in *F. oxysporum* causes aberrant and distorted septa formation in hyphae [47]. N-linked glycan modification-related proteins, besides their function in hyphal growth, also affect fungal responses to heat stress, as evidenced by mutant studies. For instance, the deletions of glucosidases (MoGls1 [21%], MoGls2 [30%], and MoGtb1 [24%]) in *M. oryzae* lead to a 20–30% decrease in mycelial growth, while calnexin mutants exhibit a 17% growth deficiency in both *M. oryzae* and *C. graminicola* [15,16]. Conversely, glucosidases (UmGls1, UmGas1, and UmGas2) and calnexin (UmCNX1) are nonessential for *U. maydis* under usual conditions (28 °C) but become vitally necessary for thermotolerance, as their deletion mutants exhibit total growth suppression at 36 °C [55,56].

### 3.2. N-Glycosylation Impacts the Developmental Processes of Plant Pathogens

The development of plant pathogens encompasses a sequence of strictly regulated biological processes, including sporulation, germination, host attachment, penetration, colonization, reproduction, and spread [63]. Notably, these diverse developmental processes critical for pathogenesis are regulated by certain *N*-glycosylation-associated proteins.

Conidia are the essential structures enabling plant pathogenic fungi to perform asexual reproduction, promote rapid diffusion, initiate the development of infectious mycelia, regulate pathogenicity and adapt to environmental stresses [64]. They are critical in the life cycle and pathogenic processes of pathogens. The morphology and development of conidia depend on *N*-glycosylation-related proteins, as evidenced by mutants deficient in N-linked glycan assembly or modification components. Certain assembly-related proteins exhibit species-specific effects in conidial differentiation. *N*-acetylglucosaminyl transferase knockout mutants exhibit pronounced conidial aggregation in *F. oxysporum* (FoGnt2) [47]. The deletions of α-1,2-mannosyltransferase results in substantial decrease in conidiation and pronounced morphological changes in *M. oryzae* (MoAlg9) [6], while they completely inhibit dimorphic switching, preventing the transition from yeast to hyphal form in *M. graminicola* (MgAlg2) [48]. In contrast, the knockout of α-1,3-mannosyltransferases do not influence germination or appressorium formation in *M. oryzae* (MoAlg3) or *C graminicola* (CgAlg3) [16,49], but it impedes infectious hyphal proliferation in *M. oryzae* (MoAlg3) [49], shortens conidial length in *M graminicola* (MgAlg3) [48] and enhances sporulation in *U. virens* (UvAlg3) [50]. The elimination of α-1,6-mannosyltransferases markedly diminishes micronucleus production in *F. oxysporum f.* sp. *cubense* (FoOch1) [51] and inhibits micronucleus formation in *V. dahlia* (VdOch1) [52]. The deletion of oligosaccharyltransferases (OST) diminishes conidial germination rates in *V. dahlia* (VdSTT3) [53] and significantly curtails sporangial release and zoospore formation in *P. capsici* (PcSTT3) [54]. Similarly, several modification-related proteins demonstrate varying effects on conidial development among different fungal species. In *M. oryzae*, glucosidases and calnexin are crucial for normal development, their deletions significantly reduce sporulation (to ≤50% of wild-type levels) and lead to sparse conidiophores with markedly fewer conidia [15]. In *U. maydis*, the aforementioned two proteins exhibit just negligible effects. The loss of glucosidase I (UmGls1) results in uneven septation in the hyphae extending to the tip of the appressorium [56]. In *C. graminicola*, calnexin is nonessential, since its mutants exhibit conidiation efficiency and appressorium formation akin to the wild-type strain [16].

Upon detecting signals from the plant host, phytopathogenic fungi generally form appressoria that provide considerable expansion pressure or release hydrolytic enzymes to penetrate plant cell wall defense, thereby initiating infection. Thereafter, these pathogens inhabit and proliferate within host cells, promoting the spread of the infection [65]. Hence, the penetration and colonization of the pathogens are essential to their pathogenicity. Certain *N*-glycosylation-associated proteins demonstrate diverse impacts on the penetration and colonization of pathogens, and they are categorized into four distinct groups based on functional disparities. Type I proteins are those that are non-essential for the penetration and colonization of pathogens. Type II proteins are exclusively necessary for the pathogen’s penetration. Type III are essential solely for the pathogen’s colonization. Type IV proteins demonstrate significance in both penetration and colonization. During the assembly of N-linked glycans, Type II proteins encompass α-1,2-mannosyltransferase from *M. graminicola* (MgAlg2) [48], α-1,6-mannosyltransferase from *F. oxysporum f.* sp. *cubense* (FoOch1) [51] and oligosaccharyltransferase from *V. dahlia* (VdSTT3) [53]; their deletions result in a complete loss of pathogens penetration. Type III proteins include α-1,3-mannosyltransferases from *M. oryzae* (MoAlg3) [49] and *U. virens* (UvAlg3) [50]; their deletions do not affect penetration but significantly inhibit fungal growth in host tissues. Type IV proteins consist of α-1,2-mannosyltransferase from *M. oryzae* (MgAlg9) [6], α-1,6-mannosyltransferase from *V. dahlia* (VdOch1) [52], α-1,3-mannosyltransferases from *C. graminicola* (CgAlg3) [16] and *N*-acetylglucosaminyl transferase from *F. oxysporum* (FoGnt2) [47]; their deletions markedly diminish pathogens penetration and colonization. The effects of modification-related proteins on the penetration and colonization of pathogens are also investigated in *U. maydis*, *M. oryzae*, and *C. graminicola*. In *U. maydis*, the proteins UmCNX and UmMns1 are classified as Type I proteins [56]; in contrast, UmGas1, UmGas2 and UmPdi1 are categorized as Type III proteins, with their deletion mutants exhibiting significant colonization defects, as their hyphal extension within host cells is severely impeded [56,57]; meanwhile, the UmGls1 protein is classified as Type IV, with its deletion entirely obstructing fungal progression within the plant during the early stages of infection [57]. In *M. oryzae*, glucosidases (MoGls1, MoGls2 and MoGtb1) and calnexin (MoCNX1) are classified as Type III, where the knockout of MoGls1, MoGls2, and MoCNX1 inhibit mycelial development within plant tissues, whereas the deletion of MoGtb1 retards invasive hyphal elongation [15]. In *C. graminicola*, calnexin (CgCNX1) is classified as Type IV proteins, crucial for the pathogen’s penetration and colonization. Its deletion leads to 80% of appressoria being unable to breach the host cell wall, while the remaining 20% display significant growth arrest within the host cell shortly after initial penetration [16].

In addition, pathogenicity of plant pathogens is influenced by *N*-glycosylation-associated proteins, as demonstrated in mutant strains lacking these specified proteins. Disruptions in glycosyltransferases implicated in N-linked glycan assembly result in diminished virulence, exhibiting different levels of pathogenicity decrease. The deletion of the oligosaccharyltransferase (VdSTT3) leads to a 30% decrease in the disease index of seedlings infected by *V. dahlia* [53]. The removals of α-1,3-mannosyltransferase (CgAlg3) and α-1,2-mannosyltransferase (MoAlg9) result in approximately an 80% reduction in leaf lesion area following infection by *C. graminicola* and *M. oryzae*, respectively [6,16]. The deletion of α-1,2-mannosyltransferase (MgAlg2) leads to a total loss of pathogenicity in *M. graminicola* [48]. Consistently, the proteins involved in N-linked glycan modification modulate pathogens’ virulence. In *U. maydis*, the glucosidases (UmGas1, UmGls1 and UmGas2) and chaperone (UmPdi1) are essential for the pathogenicity [56,57], whereas calnexin (UmCNX) and mannosidase (UmMns1) show negligible contributions [56]. Deletion mutants of UmGas1, UmGls1, or UmPdi1 exclusively cause chlorotic symptoms in infected plants, while UmGas2 deletion mutants specifically trigger anthocyanin accumulation, leading to distinct phenotypic variations compared to the tumor formation characteristic of wild-type-infected plants [56]. In *M. oryzae*, deletion mutants of glucosidases (MoGls1, MoGls2 and MoGtb1) and calnexin (MoCNX1) exhibit markedly reduced virulence [15]. The calnexin (CgCNX1) deletion mutant of *C. graminicola* entirely forfeits its ability to generate disease [16].

### 3.3. N-Glycosylation Impacts the Cell Wall Integrity of Pathogens

Fungal cell wall, primarily consisting of chitin, β-glucans, and glycoproteins, provides mechanical rigidity to endure osmotic pressure and environmental changes while also serving as a primary defense against host immune surveillance [61,65]. Plant pathogens dynamically remodel cell wall composition to evade host immune detection during invasion, thereby securing their survival, effective colonization and proliferation within the host [66]. Considering the significance of the aforementioned dynamic structure, the influence of certain *N*-glycosylation-related proteins on cell wall integrity has been summarized. Certain glycosyltransferases associated with N-linked glycan assembly are essential for the maintenance of cell wall integrity, as demonstrated in certain pathogenic fungi. The deletions of FoGnt2 (*F. oxysporum*) [47], MgAlg2 (*M. graminicola*) [48], VdOch1 (*V. dahliae*) [52], MoAlg9 and MoAlg3 (*M. oryzae*) [6,49], and UvAlg3 (*U. virens*) [50] lead to severe growth inhibition when the pathogens are exposed to cell wall stress-inducing agents such as calcofluor white (CFW), congo red (CR), sodium dodecyl sulfate (SDS) and NaCl. Notably, the deletion mutants of the initial three proteins (FoGnt2, MgAlg2 and VdOch1) demonstrate reduced protein levels and abnormal hypo-*N*-glycosylation of cell wall-associated proteins. Concurrent investigations on proteins associated with N-linked glycan modification (UmGas1 of *U. maydis* [55] and MoGls2 of *M. oryzae* [58]) demonstrate altered cell wall structure, with the MoGls2 deletion mutant exhibiting distinct susceptibility to salt, osmotic and lytic stresses, while displaying heightened sensitivity to cell wall perturbations.

### 3.4. N-Glycosylation Impacts the Immune Evasion of Pathogens Against the Host

Pathogens and host plant immune defense systems have engaged in a continuously developing arms race during their extended coevolution, resulting in the development of new pathogenic adaptations and plant counter-defenses. To counteract the virulence of pathogens, pattern recognition receptors (PRRs) on plant cell surfaces recognize pathogen-associated molecular patterns (PAMPs) [67], thereby initiating the primary defense mechanism in plants termed pattern-triggered immunity (PTI), which offers a basic level of disease resistance [62]. Nucleotide-binding/leucine-rich repeat (NLR) receptors within host cells identify pathogen effectors, thereby initiating the secondary defense mechanism known as effector-triggered immunity (ETI), which offers a more robust and enduring defense strategy [68]. To effectively infect host cells, plant pathogens employ diverse strategies to evade detection by the host cell defense systems.

PAMPs are conserved molecular structures located in the cell walls or secreted components of pathogens, including chitin, β-1,3-glucan, and mannans found in fungal cell wall, along with secreted proteins like chitinases and effectors [69,70]. Given that certain *N*-glycosylation-related proteins regulate fungal cell wall remodeling, several researchers have examined their effects on the elicitation of plant immune responses by pathogens. The N-linked glycosylation apparatus, specifically glycosyltransferases engaged in *N*-glycan assembly and glucosidases modifying N-linked glycan, serve as critical virulence determinants by suppressing host immunological responses. Infection with *F. oxysporum* deficient in FoGnt2 (*N*-acetylglucosaminyl transferase) elicits significant stimulation of host defense-related genes expression, including a 2.67-fold upregulation of GluB, 1.44-fold for Chi3, 3.20-fold for Chi9, and 3.80-fold for Pr1 [47]. Likewise, the ablation of α-1,3-mannosyltransferases (MoAlg3 and UvAlg3) in *M. oryzae* and *U. virens* results in a significant buildup of reactive oxygen species (ROS) in plants during pathogenic infection [46,47]. Furthermore, *U. maydis* mutants that disrupted glucosidase I (UmGas1) and glucosidase II (UmGls1) not only elicit ROS bursts and upregulate defense-related genes expression in infected host plants, but also trigger extensive cell death [55,56]. These findings underscore the importance of glycosyltransferases and glucosidases in plant-pathogen interactions.

Pathogens employ many strategies to successfully invade and establish themselves within a host. Among these tactics, effector proteins released by infections are crucial in inhibiting the host’s immune responses [71]. The majority of effectors are secreted by the typical endoplasmic reticulum (ER)-Golgi secretion pathway [72], functioning either outside the host cell as apoplastic effectors or within the live host cell as cytoplasmic effectors [73]. The secretion and functional stability of many apoplastic effectors are precisely regulated by *N*-glycosylation. The effector MoSlp1 from *M. oryzae*, which protects cell wall chitin from host detection, possesses three *N*-glycosylation sites (Asn48, Asn104, and Asn131) regulated by MoAlg3. The secretion and chitin-binding activities of MoSlp1 depend on simultaneous glycosylation at the aforementioned three locations. This phenomenon has been confirmed by the observation that mutations in any individual *N*-glycosylation site result in mutants that provoke significant ROS accumulation in rice; conversely, the external application of MoSlp protein glycosylated at all three sites significantly reduces the ROS burst in host cells during infection [49]. Also importantly, the functionality of the xyloglucan-specific endoglucanase PsXEG1 from *P. sojae* is contingent upon *N*-glycosylation. PsXEG1, a cell wall-degrading protein in soybean, contains two *N*-glycosylation sites (Asn174 and Asn190). These modifications not only obstruct the binding and degradation of the host apoplastic aspartic protease (GmAP5) to PsXEG1, but also diminish the binding of host inhibitors GmGIP1. Mutations in the aforementioned two glycosylation sites result in diminished biomass of *P. sojae* that effectively infected hypocotyls, yielding an approximate 35% reduction in total pathogenicity [74]. Furthermore, the dimerization and abundance of the effector LtScp1 from *Lasiodiplodia theobromae* are modulated by *N*-glycosylation. LtScp1 possesses three *N*-glycosylation sites, which significantly safeguards the chitin component in fungal hyphae from hydrolysis by the host-derived chitinase VvChi4 [75]. Moreover, the effector protein VdSCP23, secreted by *V. dahlia*, contains two *N*-glycosylation sites that are crucial for *V. dahlia* in effectively inhibiting various host immune responses, including programmed cell necrosis, reactive oxygen species (ROS) burst, plasma membrane electrolyte leakage, and the transcriptional activation of defense-related genes [76]. The secretion of virulence factors in *U. maydis*, especially effector proteins that facilitate host–pathogen interactions, need effective *N*-glycosylation of the disulfide isomerase Pdi1, which catalyzes disulfide bond formation [57]. The secretion and stability of the *C. graminicola* effectors necrosis-inducing secreted protein 1 (CgNIS1) and biotrophy-associated secreted protein 3 (CgBAS3) are similarly controlled by CgAlg3 and CgCnx1. This regulation is evidenced by CgAlg3-deficient mutants, which display significantly reduced levels of these effectors, and CgCnx1-deficient mutants, which show accelerated degradation of CgNIS1 and CgBAS3 upon treatment with the translation inhibitor cycloheximide [16].

## 4. Conclusions and Future Prospects

**Figure 3:**
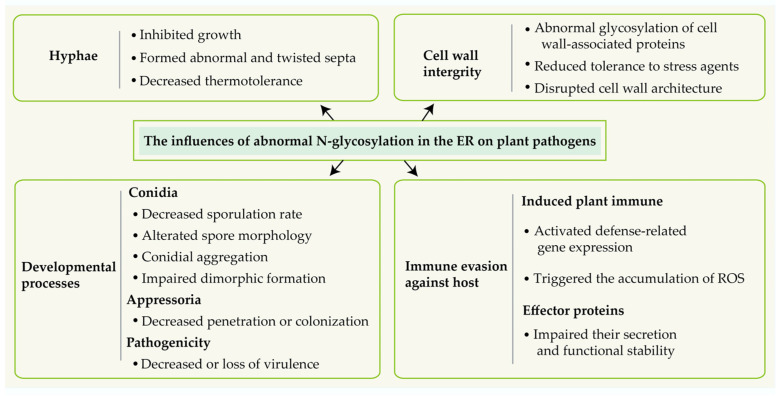
The effect of abnormal *N*-glycosylation in the endoplasmic reticulum on plant pathogens.

Mounting evidence substantiates that *N*-glycosylation is essential for the physiology and virulence processes of plant pathogens, its abnormal disrupts cell wall construction, fungal morphogenesis, the formation of infection structures, the secretion of effector protein, and the suppression of host immune responses (Figure 3). Nonetheless, the intricate mechanism of this posttranslational modification in fungal pathogenesis requires additional investigation. The current comprehension of the influence of *N*-glycosylation on the development and pathogenicity of plant pathogens is predominantly derived from functional genetic analyses of the components of *N*-glycosylation pathways, whereas the downstream *N*-glycosylated target proteins that perform specific biological functions have rarely been characterized. During the various developmental phases of plant pathogens, specific glycosyltransferase genes demonstrate stage-specific expression. For instance, in *F. graminearum*, *ALG5* and *GLS1* show markedly increased transcript levels during vegetative growth, while *ALG12* exhibits enhanced transcription exclusively during sexual developmental stages [77]. This variability contributes to the differences in *N*-glycan structures and glycosylation sites of target proteins across different developmental stages. To date, systematic investigations on *N*-glycosylation targets have predominantly concentrated on a limited number of typical plant pathogens, including *M. oryzae* [15], *C. graminicola* [16], *F. graminearum* [77] and *P. sojae* [17]. Given the species-specific distribution of *N*-glycosylation-related proteins, comparative investigations involving other plant pathogenic fungus species are needed. In future research, the comprehensive glycomics investigation employing integrated glycomics and proteomics approaches, and the clarification of their functional processes through molecular genetics methodologies will be crucial to elucidate the regulatory mechanisms of *N*-glycosylation in plant pathogens. These studies will substantially enhance the accurate identification of pathogenicity-determining targets, providing a robust scientific foundation for the development of environmentally benign and highly effective fungicidal medicines that precisely target *N*-glycosylated proteins.

## Figures and Tables

**Table 1:** The *S. cerevisiae* proteins involved in *N*-glycosylation assembly and ER-mediated modification along with their functions and products.

Classes	Location	Enzymes		Function	Product
Assembly of N-linked glycan	ER cytoplasm	Mpg1 (Alpha-D-mannose-1-phosphate)		it was involved in the synthesis of GDP-mannose from GTP and mannose-1-phosphate	GDP-mannose
		Sec59 (Dolichol kinase)		it was responsible for the synthesis of dolichol phosphate (Dol-P)	Dol-P
		Dpm1 (Dolichol phosphate mannose synthase)		it catalyzed the transfer of mannose from GDP-mannose to dolichol phosphate.	Dol-P-mannose (Dol-P-Man)
		Alg5 (Dolichol phosphate glucose synthase)		it catalyzed the transfer of glucose from UDP-glucose to dolichol phosphate.	Dol-P-glucose (Dol-P-Glc)
		Glycosyltransferases	Alg7 (*N*-acetylglucosamine-phosphate transferase)	it can add GlcNAc-P to Dol-P, forming the anhydride dolichylpyrophosphate-GlcNAc (Dol-PP-GlcNAc)	Dol-PP-GlcNAc
			Alg13/Alg14 (Beta-1,4-*N*-acetylglucosaminyltransferase)	it transfers the second GlcNAc residue to Dol-PP-GlcNAc	Dol-PP-GlcNAc_2_
			Alg1 (Beta-1,4-mannosyltransferase)	it adds the first Man to Dol-PP-GlcNAc_2_	Dol-PP-GlcNAc_2_-Man
			Alg2 (Alpha-1,3/1,6-mannosyltransferase)	it catalyzes addition of the two branching mannoses to Dol-PP-GlcNAc_2_-Man	Dol-PP-GlcNAc_2_-Man_3_
			Alg11 (Alpha-1,2-mannosyltransferase)	it can add two mannoses in sequence to Dol-PP-GlcNAc_2_-Man	Dol-PP-GlcNAc_2_-Man_5_
		Rft1 (Flippase)		it assists in the transmembrane translocation of the glycolipid	Dol-PP-GlcNAc_2_-Man_5_
	ER Lumen	Glycosyltransferases	Alg3 (Alpha-1,3-mannosyltransferase)	the addition of an α-1,3-linked mannose to Dol-PP-GlcNAc_2_-Man_5_	Dol-PP-GlcNAc_2_-Man_6_
			Alg9 (Alpha-1,2-mannosyltransferase)	the addition of an α-1,2-linked mannose to Dol-PP-GlcNAc_2_-Man_6_	Dol-PP-GlcNAc_2_-Man_7_
			Alg12 (Alpha-1,6-mannosyltransferase)	the addition of an α-1,6-linked mannose to Dol-PP-GlcNAc_2_-Man_7_	Dol-PP-GlcNAc_2_-Man_8_
			Alg9 (Alpha-1,2-mannosyltransferase)	the addition of an α-1,2-linked mannose to Dol-PP-GlcNAc_2_-Man_8_	Dol-PP-GlcNAc_2_-Man_9_ (LLO)
			Alg6 (Alpha-1,3-mannosyltransferase)	it initiates the glucosylation of the a-antenna of the oligosaccharide	Dol-PP-GlcNAc_2_-Man_9_Glc
			Alg8 (Alpha-1,3-mannosyltransferase)	it adds the second α-1,3-linked Glc residue to the LLO	Dol-PP-GlcNAc_2_-Man_9_Glc_2_
			Alg10 (Alpha-1,2-mannosyltransferase)	it adds the third α-1,2-linked Glc residue to the LLO	Dol-PP-GlcNAc_2_-Man_9_Glc_3_
		Oligosaccharyltransferase (OST)	OST1, OST5	it catalyzes the transfer of the oligosaccharide from the lipid carrier dolichylpyrophosphate to the amide group of selected asparagine residues of polypeptide chain	Asn-GlcNAc_2_-Man_9_Glc_3_
			Stt3, Ost4, Ost3/Ost6		
			Ost2, Wbp1, Swp1		
Proceeding of N-linked glycan (ER)		Gls1 (Glucosidase I)		it cleaves the terminal α-1,2-linked glucose residues from the Glc_3_Man_9_GlcNAc_2_ oligosaccharide	Asn-GlcNAc_2_-Man_9_Glc_2_
		Gls2 (Glucosidase II)		it removes the first α-1,3-linked glucose residues from the Glc_2_Man_9_GlcNAc_2_ oligosaccharide	Asn-GlcNAc_2_-Man_9_Glc
		CNX1/CRT1 (Calnexin/calreticulin)		bound specifically to monoglucosylated proteins	Asn-GlcNAc_2_-Man_9_Glc
		Gls2 (Glucosidase II)		it removes the second α-1,3-linked glucose residues from the Glc_2_Man_9_GlcNAc_2_ oligosaccharide	Asn-GlcNAc_2_-Man_9_
		Mns1 (Alpha-1,2-mannosidase)		it removes one specific mannose	Asn-GlcNAc_2_-Man_8_
		UGGT1 (UDP-glc: glycoprotein glucosyltransferase 1)		it reglucosylated the non-native unglucosylated proteins to decides whether glycoproteins traffic onto the Golgi and beyond, or are retained in the ER for further assistance	Asn-GlcNAc_2_-Man_9_Glc

**Table 2:** The impact of the absence of *N*-glycosylation-related proteins on the phenotype in plant pathogens.

Category	Fungus Species	Protein	Molecular Function	Phenotypic Impacts of Protein Knockout						Res
				Ve	Cd	Pe and Co	Pa	CWI	Im	
Assembly-related proteins	*Fusarium oxysporum*	FoGnt2	*N*-Acetylglucosaminyl transferase	aberrant twisted septa	conidial aggregation	significant reduction in pe and co (Type IV)	dramatic reduction	high sensitivity to SDS and CFW	induced significant upregulation of defense-related genes	[47]
	*Mycosphaerella graminicola*	MgAlg2	a-1,2-Mannosyltransferase	no reported	yeast-to-hypha conversion defect	complete loss in pe (Type II)	complete absence of pathogenicity	high sensitivity to CFW, abnormally hypo-*N*-glycosylated proteins	no reported	[48]
	*Magnaporthe oryzae*	MoAlg9	α-1,2-mannosyltransferase	no impact	decreased sporulation and morphological changes	significant reduction in pe and co (Type IV)	significantly reduced virulence	high sensitivity to NaCl, sorbitol, and KCl	no reported	[6]
		MoAlg3	a-1,3-Mannosyltransferase	growth retardation (6% reduction)	inhibited growth of infectious hyphal	severely inhibited fungal growth in host tissues (Type III)	80% reduction in leaf lesion area	high sensitivity to CFW, CR, and SDS	induced ROS burst	[49]
	*Colletotrichum graminicola*	CgAlg3		growth retardation (29% reduction)	reduced conidial length	significant reduction in pe and co (Type IV)		no reported	no reported	[16]
	*Ustilaginoidea virens*	UvAlg3		growth retardation (30% reduction)	increased sporulation	severely inhibited fungal growth in host tissues (Type III)	significantly reduced virulence	high sensitivity to sorbitol and NaCl	induced ROS burst	[50]
	*Fusarium oxysporum f. sp. cubense*	FoOch1	a-1,6-mannosyltransferase	growth retardation (60% reduction)	fewer microconidia formation	complete loss of pe (Type II)	complete loss of pathogenicity	a reduced amount of cell wall proteins	no reported	[51]
	*Verticillium dahlia*	VdOch1			absence of microsclerotia formation	significant reduction in pe and co (Type IV)	significantly reduced virulence	high sensitivity to SDS and CR		[52]
		VdSTT3	Oligosaccharyltransferase	growth retardation (30% reduction)	decreased conidial germination	complete loss of pe (Type II)	30% reduction in seedling disease index	no reported		[53]
	*Phytophthora capsici*	PcSTT3			reduced sporangial release and zoospore production	no reported	significantly reduced virulence			[54]
Modification-related proteins	*Ustilago maydis*	UmGls1	Glucosidase I	compromised growth at 36 °C	irregulated septation patterns	blocked early infection (Type IV)	chlorosis in infected plants	alterations in cell wall components	induced ROS burst, defense gene upregulation, extensive cell death	[55]
		UmGas1	Glucosidase II		no impact	severe inhibition of hyphal extension in host cells (Type III)		no reported		[56]
		UmGas2	Glucosidase II β-subunit				anthocyanin accumulation		no impact	
		UmCNX1	Calnexin			no impact (Type I)	no impact	no impact		
		UmMns1	Mannosidase	no impact						
		UmPdi1	Disulfide isomerase			severe inhibition of hyphal extension in host cells (Type III)	chlorosis in infected plants			[57]
	*Magnaporthe oryzae*	MoGls1	Glucosidase I	growth retardation (21% reduction)	decreased conidiation, sparse conidiophores bearing fewer conidia	inhibited mycelial development within host (Type III)	significantly reduced virulence	no reported	no reported	[15]
		MoGls2	Glucosidase II	growth retardation (30% reduction)				decreased tolerance to salt stress, osmotic stress, and lytic agents; enhanced susceptibility to cell wall damage		[15,58]
		MoCnx1	Calnexin	growth retardation (17% reduction)				no reported		[15,58]
		MoGtb1	Glucosidase II β-subunit	growth retardation (24% reduction)		decelerated invasive hyphal elongation (Type III)				
	*Colletotrichum graminicola*	CgCnx1	Calnexin	growth retardation (17% reduction)	no impact	80% appressoria penetration failure and 20% growth arrest within host (Type IV)	complete loss of pathogenicity			[16]

Ve: Vegetative hypha growth; Cd: Conidial development; Pe and Co: Penetration and colonization; Pa: Pathogenicity; CWI: Cell wall integrity; Im: Immune evasion against host; Res: References; ROS: reactive oxygen species.

## Data Availability

No new data were created or analyzed in this study. Data sharing is not applicable to this article.
